# Meta-analysis of the efficacy of treatments for newly diagnosed and relapsed/refractory multiple myeloma with del(17p)

**DOI:** 10.18632/oncotarget.18722

**Published:** 2017-06-27

**Authors:** Jinghua Liu, Hui Yang, Xiaochan Liang, Yuxin Wang, Jian Hou, Yanqin Liu, Jigang Wang, Fan Zhou

**Affiliations:** ^1^ Department of Hematology, The General Hospital of Shenyang Military, Shenyang, China; ^2^ Department of Clinical Medicine, Shenyang Pharmaceutical University, Shenyang, China; ^3^ Department of Hematology, The Myeloma and Lymphoma Center, Chang Zheng Hospital, The Second Military Medical University, Shanghai, China

**Keywords:** multiple myeloma del(17p), carfilzomib, pomalidomide, lenalidomide, bortezomib

## Abstract

We analyzed the treatment of newly diagnosed and relapsed/refractory multiple myeloma (NDMM/RRMM) patients with del(17p). Thirteen prospective studies that evaluated 3,187 MM patients, including 685with del(17p), were included in our meta-analysis. The incidence of del(17p) in NDMM and RRMM patients was similar (13% vs. 14%, respectively, *P* = 0.64, *I*^2^ = 94%). The overall response rate (ORR) to new agents was 40.5% and 67.1%, respectively, in RRMM patients with or without del(17p) (*P =* 0.1, *I*^2^ = 63.9%). NDMM patients with del(17p) treated with PAD (bortezomib, adriamycin, and dexamethasone) induction therapy followed by bortezomib maintenance therapy had higher progression-free survival (PFS) (25.7 vs. 12-14.6 months) and overall survival (OS) (62% vs. 8% at 36 months) than those treated with PD (bortezomib and dexamethasone) or VAD (vincristine, adriamycin, and dexamethasone). PFS among RRMM patients with del(17p) treated with D (single-agent dexamethasone), Rd/VRd (lenalidomide and dexamethasone/bortezomib and Rd), KRd (carfilzomib and Rd), IRd (ixazomib and Rd), ERd (elotuzumab and Rd), or P+D (pomalidomide and dexamethasone) was 1.1, 2-14.9, 24.5, 15.7, 21.2, and 4.6-7.3 months, respectively. The OS of patients treated with D or K (single-agent carfilzomib), Rd/VRd, ERd, or P+D was 7.7, 7, 4.7–36.4, > 42.3, and 12–12.6 months, respectively. PFS among RRMM patients without del(17p) treated with D, Rd/VRd, ERd, or P+D was 2.3, 8.2-14.8, 18.5, and 4.2 months, while OS was 9, 23-40.8, 42.3, and 14 months, respectively. Thus bortezomib maintenance therapy improves the prognosis of NDMM patients with del(17p). Combined treatment with carfilzomib or elotuzumab and Rd, or pomalidomide with low-dose dexamethasone, improves the outcomes of RRMM patients with del(17p).

## INTRODUCTION

Multiple myeloma is a heterogeneous B-cell malignancy. The median overall survival (OS) is 2–10 or more years and is dependent upon host factors, tumor burden, cytogenetic abnormalities (CAs), and therapeutic response [1–3]. High-risk CAs include del(17p), t(4;14), t(14;16), and t(14:20) [2, 4]. The frequency of t(14;20) is 1%. The adverse impact of t(14:16) can be abrogated by double autologous hematopoietic stem cell transplantation (auto-HSCT) [5]. Bortezomib improves the prognosis of patients with t(4;14). Tandem auto-HSCT prolonged survival in patients with t(4;14) [6–7]. TP53 (chromosome 17p) is deleted in 7% of myelomas. TP53 deletion induces clonal immortalization and promotes tumor cell survival. The presence of del(17p) is associated with reduced OS in myeloma patients, despite treatment with proteasome inhibitors and immunomodulatory drugs [8].

Bortezomib is a first generation proteasome inhibitor. Several randomized trials have evaluated bortezomib for induction, consolidation, or maintenance therapy in MM patients with del(17p). Bortezomib/dexamethasone did not improve the outcomes of patients with del(17p) in IFM-2005-01 [9]. In contrast, bortezomib-based induction and maintenance therapy improved the outcomes of del(17p) patients in HOVON65/GMMG-HD4. However, OS was lower in these patients compared to those without del(17p) [10]. Lenalidomide and pomalidomide are immunomodulatory drugs. There is limited data on the effectiveness of lenalidomide as a first-line therapy for MM patients with del(17p). Treatment of relapsed/refractory multiple myeloma (RRMM) patients with del(17p) with lenalidomide alone or in combination with dexamethasone (Rd) has shown inconsistent results [11–13, 18]. The combination of carfilzomib or elotuzumab and Rd improved the outcomes of del(17p) patients [13, 22]. The same results were observed in RRMM patients with del(17p) treated with pomalidomide/dexamethasone [14]. We performed a meta-analysis of 13 studies to investigate the treatment of RRMM patients with del(17p) in order to determine the optimal therapeutic regimen.

## RESULTS

### Study characteristics

**Figure 1:**
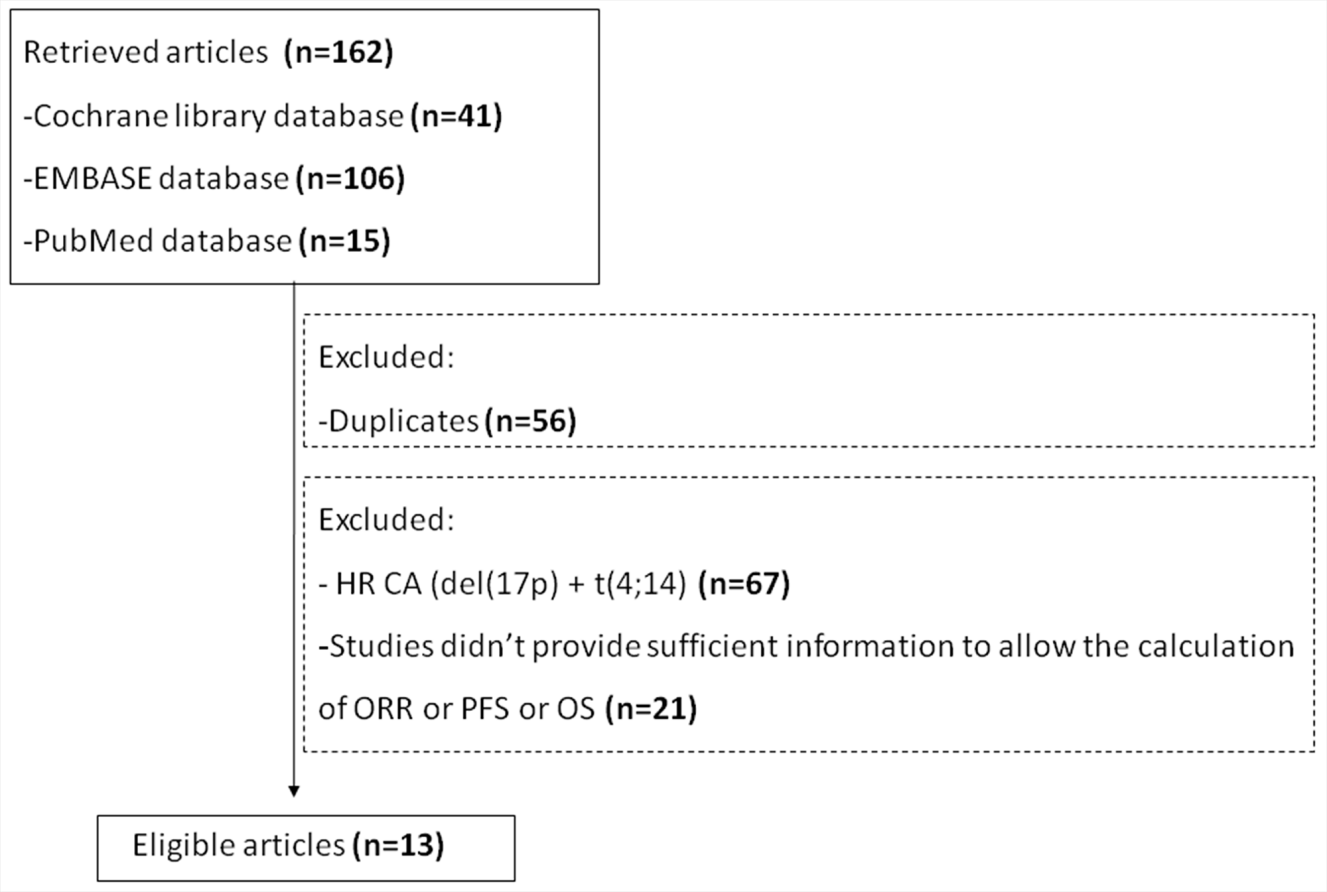
Flowchart of study inclusion

**Figure 2:**
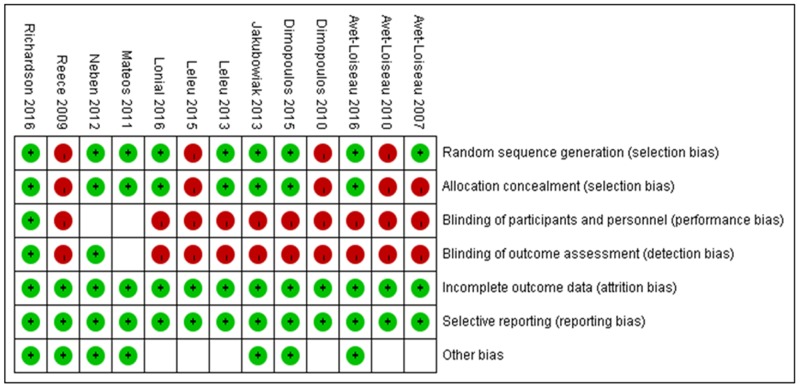
Quality of the studies Green circle represents low risk, red circle represents high risk and white square represents unclear risk.

Overall, 162 studies were retrieved using the search strategy. Thirteen were included in the meta-analysis [9–14, 16–22]. The exclusion criteria are shown in Figure 1. The study characteristics are shown in Tables 1–2. One study reported the outcomes of NDMM patients with del(17p) treated with VAD (vincristine, adriamycin, and dexamethasone), three reported the outcomes of bortezomib combination regimens in NDMM patients with del(17p), three reported the outcomes of lenalidomide combination regimens in RRMM patients with del(17p), three reported the outcomes of pomalidomide combination regimens in RRMM patients with del(17p), and three reported the outcomes of carfilzomib combination regimens in RRMM patients with del(17p) (Figure 2).

**Table 1:** NDMM study characteristics

Author, year	Age (M)	Regimen	Del(17)			No Del(17)		
			n	PFS(m)	OS (m)	n	PFS(m)	OS (m)
Avet-Loisea 2007 [17]	49	4VAD + auto-HSCT	58	14.6	22.4	474	34.7	75% at 41 m
Avet-Loisea 2010 [9]	57	4PD + auto-HSCT	54	14	50% at 48 m	453	36	79% at 48 m
		4 VAD + auto-HSCT	119	14 or so	50% or so at 48 m	393	-	-
Neben 2012 [10]	57	3 PAD + auto-HSCT + bortezomib maintenance 1/2 w for 2 years	37	25.7	62% at 36 m	313	36	85% at 36 m
		3 VAD + auto-HSCT + thalidomide maintenance 50 mg for 2 years		12	8% at 36 m			80% at 36 m
Mateo 2011 [16]	73	VMP one 6-week + five 5-week + VP maintenance up to 3 years	24	24	27	208	33	75% at 36 m
		VTP one 6-week + five 5-week + VT maintenance up to 3 years						

VAD, vincristine, adriamycin, and dexamethasone; PD, bortezomib and dexamethasone; PAD, bortezomib, adriamycin, and dexamethasone; VMP, bortezomib, melphalan, and prednisone; VP, bortezomib and prednisone; VTP, bortezomib, thalidomide, and prednisone; VT, bortezomib and thalidomide; auto-HSCT autologous hematopoietic stem cell transplantation.

**Table 2:** RRMM study characteristics

Author, year regimen	Group	n	TFD (Y) (M)	NPT	Bor %	Lena %	Thal %	Tran %	ORR	PFS (m)	OS (m)
Reece 2009 R+/-D [11]	del(17p13)	12	1.84	≥ 3, 75%	50	0	66.7	75	58.3%	2.2	4.7
	no del(17p13)	118	3.63	≥ 3, 47%	44.9	0	52.5	72	85.6%	8.2	23.7
Jakubowiak 2013 K [19]	del(17p13)	30	5.3	5 (2-12)	100	100		72.6	16.7%	-	7
	no del(17p13)	199							26.1%	-	-
Avet-Loiseau 2016 KRd [12]	del(17p13)	13	-	2.0 (1-4)	81.3	27.1	-	79.2	76.9%	24.5	-
	no del(17p13)	182							89%	-	-
Rd	del(17p13)	13	-	2.0 (1-3)	67.3	23.1	-	59.6	46.2%	11.1	-
	no del(17p13)	209							71.8%	-	-
Dimopoulos 2010 Rd [18]	del(17p13)	3	2.8	≥ 3, 48%	76	0	76	0	0	2	9
	no del(17p13)	47							63%	9.9	23
VRd	del(17p13)	7	3	≥ 3, 46%	80	0	88	0	29%	3	9
	no del(17p13)	42							73%	9.9	23
Leleu 2013 P+D [21]	del(17p13)	15	5.9	≥ 6, 23%	100	100	73	81	33%	-	-
	no del(17p13)	50							44%	-	-
Leleu 2015 P+D [20]	del(17p13)	22	3	≥ 3, 60%	96	100	-	78	32%	7.3	12
	no del(17p13)	30							-	-	-
Doimopoulos 2015 P+D [14]	del(17p13)	44	5.3	5 (2-14)	-	-	-	-	31.8%	4.6	12.6
	no del(17p13)	258							-	4.2	14
D	del(17p13)	23	6.1	5 (2-17)	-	-	-	-	4.3%	1.1	7.7
	no del(17p13)	130							-	2.3	9.0
Richardson 2016 IRd [22]	del(17p13)	33	-	-	-	-	-	-	-	15.7	-
	no del(17p13)	519							-	-	-
Lonial 2016 ERd [13]	del(17p13)	102	-	≥ 3, 16%	68	5	48	52	-	21.2	NR
	no del(17p13)	213	-						-	18.5	42.3
Rd	del(17p13)	104	-	≥ 3, 16%	71	6	48	57	-	14.9	36.4
	no del(17p13)	218	-						-	14.8	40.8

TFD, time from diagnosis; NPT, number of prior therapies; Bor, bortezomib; Lena, lenalidomide; Thal, thalidomide; Tran, stem cell transplantation; ORR, overall response rate; NR, not reached.

### Incidence of del(17p) in NDMM and RRMM patients

**Figure 3:**
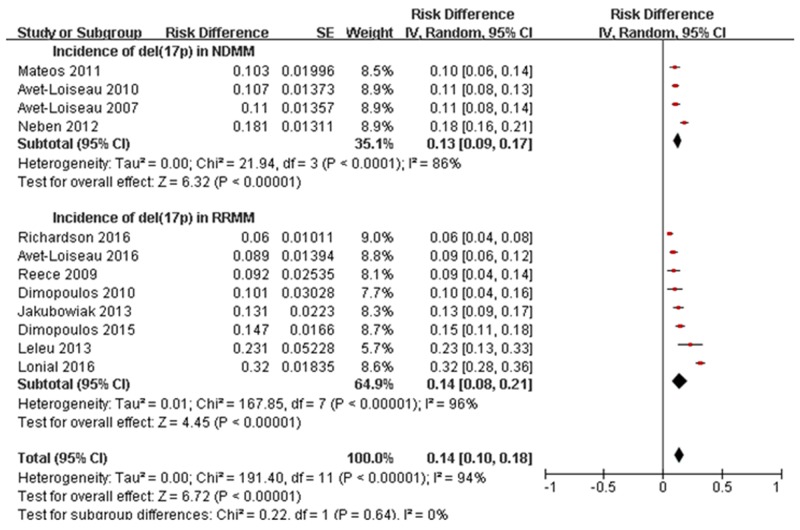
Meta-analysis of incidence of del(17p) in NDMM and RRMM

The incidence of NDMM with del(17p) was 10.3–18.1% in four studies. The pooled incidence of del(17p) was 13% (95% CI: 9–17%). There was significant study heterogeneity (*I*^2^ = 86%, *P* < 0.0001) (Figure 3). The total incidence of RRMM with del(17p) was 6–32% in eight studies. The Leleu et al. study was excluded because only del(17p) and t(4;14) MM patients were included. The pooled incidence of del(17p) was 14% (95% CI: 8–21%) with significant study heterogeneity (*I*^2^ = 96%, *P* < 0.00001) (Figure 3). The incidence of del(17p) did not differ between NDMM and RRMM patients (*P* = 0.64, *I*^2^ = 94%).

### Response rate to therapy in RRMM patients with and without del(17p)

**Figure 4:**
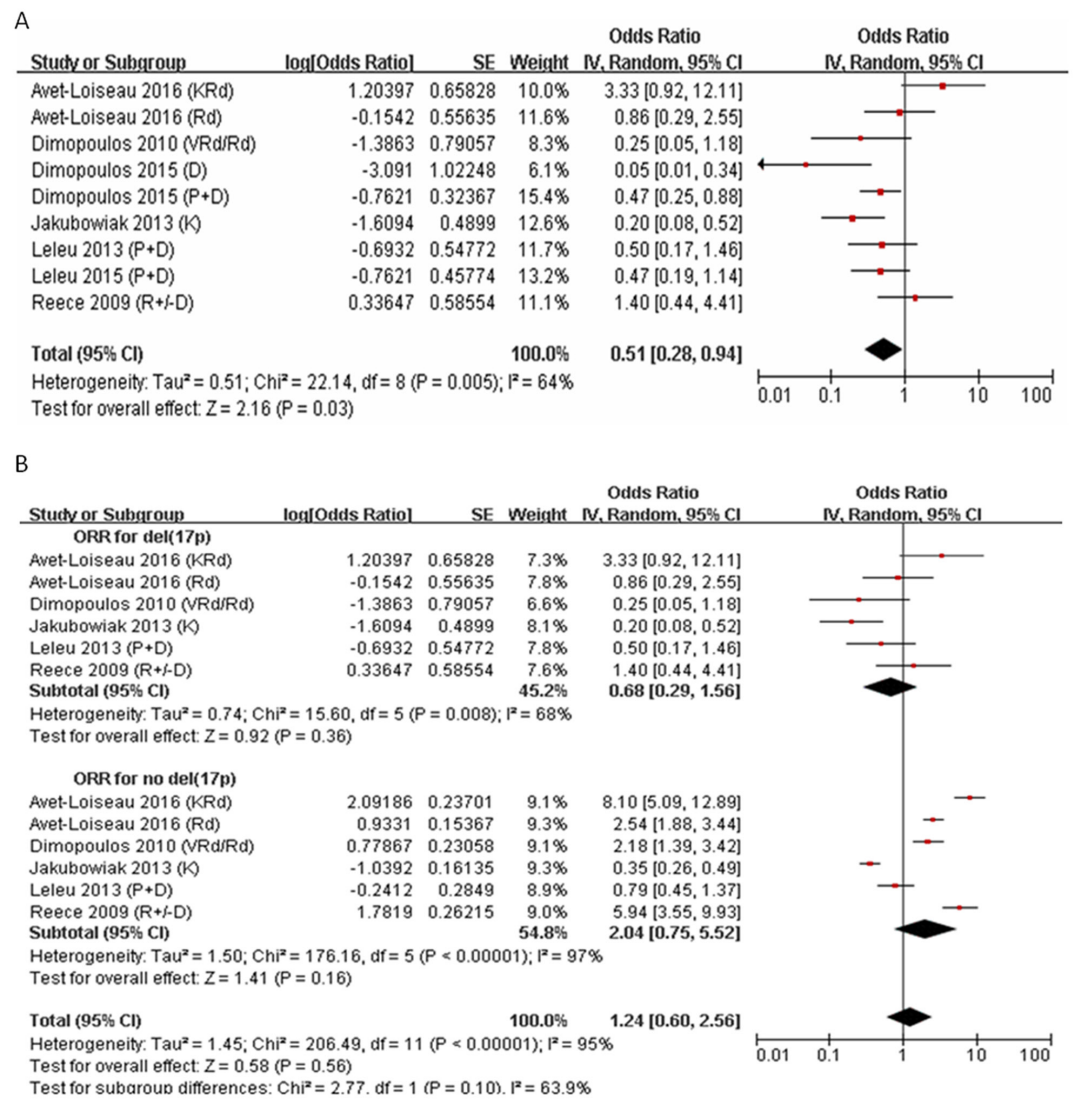
Meta-analysis of overall response rate (ORR) in RRMM with del(17p) or no del(17p) **(A)** ORR to all regimen in RRMM with del(17p). **(B)** ORR to new agents in RRMM with del(17p) or no del(17p). ORR, over response rate; CI, confidence interval.

Seven trials that enrolled a total of 1,447 RRMM patients (including 182 patients with del(17p)) evaluated the effects of treatment on ORR. The regimen included D (single-agent dexamethasone), Rd/RD (lenalidomide and dexamethasone), VRd (bortezomib and Rd), K (single-agent carfilzomib), KRd (carfilzomib and Rd), and P+D (pomalidomide and dexamethasone). The ORR to D was 4.3% in patients with del(17p) in one study. The ORR to K was 16.7% in one study. The ORR to Rd was variable in three studies (0%, 46.2%, and 58.3%, respectively). The ORR to P+D was approximately 32% (indicating efficacy) in three studies, while the ORR to KRd was 76.9% in one study (Figure 4A). We compared the ORR to new agents in 86 patients with, and 805 patients without, del(17p). Pooled analysis showed that the ORR was 40.5% (95% CI: 22.5–60.9%) in patients with and 67.1% in patients without del(17p) (95% CI: 42.9–84.7%) (*P =* 0. 1, *I*^2^ = 63.9%) (Figure 4B).

### Survival of NDMM and RRMM patients with and without del(17p)

**Figure 5:**
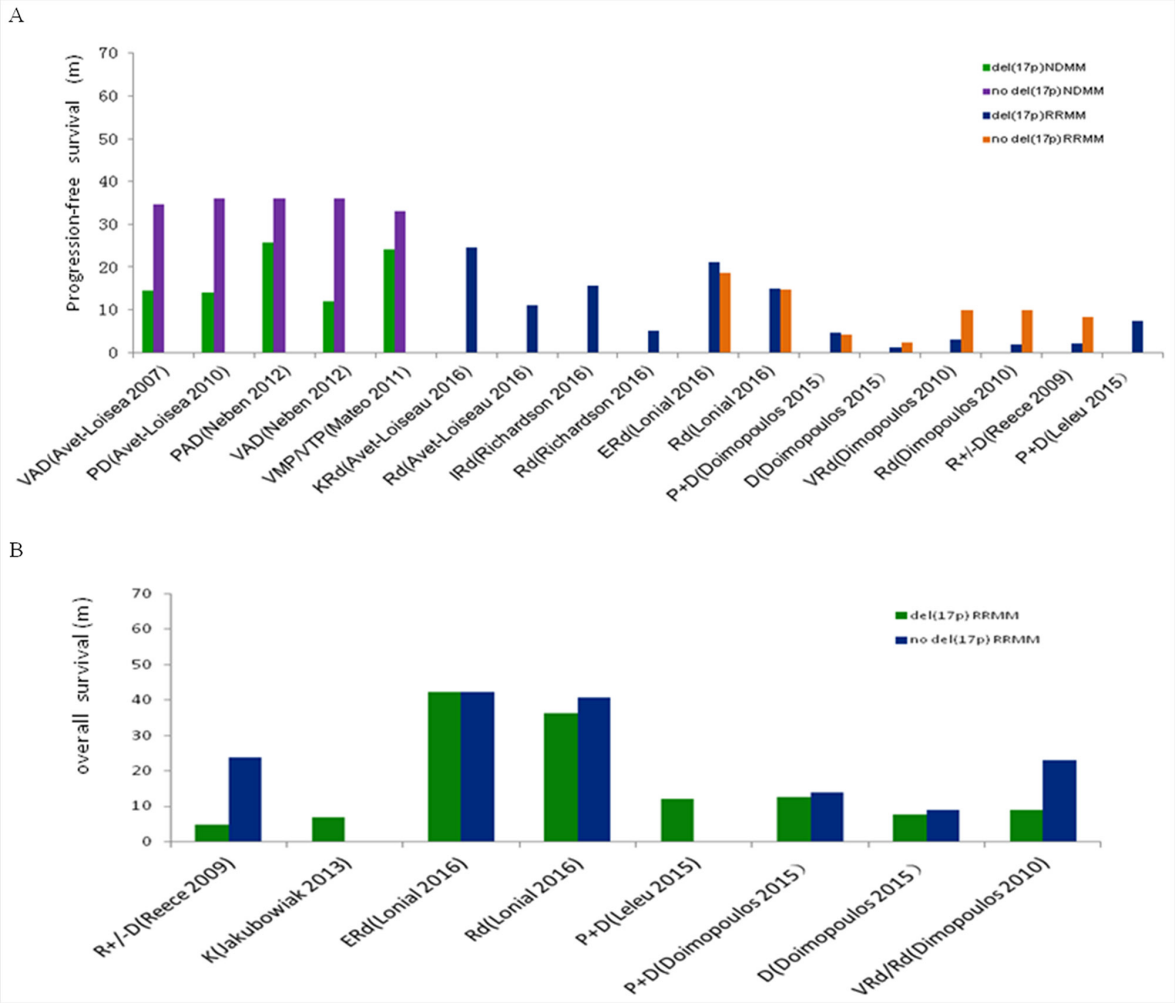
Progression-free survival (PFS) and over survival (OS) of NDMM or RRMM with del(17p) or no del(17p) **(A)** PFS of NDMM or RRMM with del(17p) or no del(17p). **(B)** OS of RRMM with del(17p) or no del(17p). VAD, vincristine adriamycin and dexamethasone; P+D, pomalidomide and dexamethasone; PAD, bortezomib adriamycin and dexamethasone; VMP, bortezomib melphalan and prednisone; VP, bortezomib and prednisone; VTP, bortezomib thalidomide and prednisone; K, carfilzomib; Rd, lenalidomide and dexamethasone; I, ixazomib; E, elotuzumab.

PFS was evaluated in four trials: three that enrolled young patients following VAD or PD/PAD induction therapy and auto-HSCT, and one that enrolled older patients who were only treated with VMP/VTP. OS was evaluated in 1,740 NDMM patients (292 with del(17p) and 1,448 without del(17p)) (Figure 5A). PFS and OS were longer in del(17p) patients treated with PAD induction therapy followed by long-term bortezomib maintenance therapy compared to patients treated with PD or VAD induction therapy without bortezomib maintenance therapy (25.7 vs. 12–14.6 months, and 62% vs. 8% at 36 months, respectively).

We evaluated PFS (Figure 5A) and OS (Figure 5B) in seven trials that included 1,419 RRMM patients (393 with and 1,026 without del(17p)). The PFS of del(17p) patients treated with D, Rd/VRd, KRd, IRd (ixazomib and Rd), ERd (elotuzumab and Rd), or P+D was 1.1, 2–14.9, 24.5, 15.7, 21.2, and 4.6–7.3 months, respectively. The OS of patients treated with D or K, Rd/VRd, ERd, or P+D was 7.7, 7, 4.7–36.4, > 42.3, and 12–12.6 months, respectively. In del(17p)-negative patients, the PFS of D, Rd/VRd, ERd, and P+D was 2.3, 8.2–14.8, 18.5, and 4.2 months, respectively. The OS of D, Rd/VRd, ERd, and P+D was 9, 23–40.8, 42.3, and 14 months, respectively.

## DISCUSSION

The incidence of del(17p) in NDMM and RRMM patients was similar (13–14%). Previous studies have demonstrated that del(17p) is a secondary genetic event in MM, while immunoglobulin heavy chain (IgH) translocation is a primary event. Late events are generally indicative of more aggressive disease. Of patients with IgH translocations, 62% had 1q gain compared to 32.4% in controls [23]. The frequency of del(17p) was similar to that of patients without IgH translocations. Among patients with IgH translocation and/or 1q gain, or del(17p), 20% shared ≥ 2 CAs [1]. We investigated the treatment of NDMM and RRMM patients with del(17p). We selected studies with data for del(17p) only (not for high-risk CAs: del(17p) and t(4;14)). The cut-off value for the proportion of plasma cells with del(17p) was ≥ 60%.

Pineda-Roman et al. [24] and Shaughnessy et al. [8] reported that the introduction of bortezomib in the Total Therapy 3 program improved the prognosis of patients with t(4;14)or del(17p). However, two other studies reported conflicting results, which may be explained by differences in study size, therapeutic strategy, or the number of bortezomib treatments. In the IFM experience, only four cycles (16 injections) of bortezomib were administered. In the HOVON-65/GMMG-HD4 [10], GEM05MAS65 [16], and Total Therapy 3 trials, patients received more than 64 injections of bortezomib. It is possible that only long-term bortezomib maintenance therapy can improve the poor prognosis of del(17p) patients. HOVON-65/GMMG-HD4, a prospective randomized controlled trial, demonstrated an improvement of PFS and OS in patients who received long-term treatment with bortezomib compared to controls (25.7 vs. 12 months, and 62% vs. 8% at 36 months, respectively). However, the trial sample size was small, and the prognosis of del(17p) patents was still worse than that of patients without del(17p).

The presence of del(17p) in RRMM patients is associated with lower treatment response rates and reduced PFS and OS. The detrimental effect of del(17p) was demonstrated in patients treated with lenalidomide and dexamethasone combination therapy, with or without the addition of bortezomib [11, 18]. Lonial et al. [13] reported higher PFS and OS, which could be explained by the fact that 49% of their patients received one previous therapeutic regimen. There is a need for novel therapeutic approaches for this highly refractory patient subgroup. Pomalidomide has anti-myeloma and immunomodulatory effects. It also inhibits stromal cell adhesion. The MM-003 and IFM2010-02 trials of pomalidomide in combination with low-dose dexamethasone found that combination therapy improved the outcomes of RRMM patients with del(17p) [14, 20]. However, patients in the IFM2010-02 study had early relapse.

Carlzomib is an epoxyketone proteasome inhibitor that binds selectively and irreversibly to the immunoproteasome. Carlzomib as a single-agent had an encouraging ORR of 16.7% in del(17p) patients [19]. The ORR was 76.9% in patients treated with carfilzomib, lenalidomide, and dexamethasone. An improvement in PFS (24.5 months) was also observed, which was a 9-month improvement compared to patients treated with lenalidomide and dexamethasone [12]. However, the study size was relatively small. In the TOURMALINE-MM1 study of ixazomib (a proteasome inhibitor), lenalidomide, and dexamethasone compared to Rd in patients with del(17p), the PFS HRs favored ixazomib, lenalidomide, and dexamethasone vs. Rd [22].

Elotuzumab is a first-in-class humanized immunoglobulin G1 immuno-stimulatory monoclonal antibody targeting SLAMF7/CS1. In the ELOQUENT-2 study, PFS and OS were longer in RRMM patients with del(17p) treated with elotuzumab, lenalidomide, and dexamethasone compared to those treated with Rd, and were even longer than in patients without del(17p) [13]. Elotuzumab has a dual mechanism of action. It promotes antibody-dependent, cell-mediated cytotoxicity, and directly activates natural killer cells through SLAMF7 receptors in an Fc-independent process. Elotuzumab activates CD16-deficient natural killer cells (CD16 is required for antibody-dependent, cell-mediated cytotoxicity), which further supports the direct immunotherapeutic role of elotuzumab in activating natural killer cells [25].

Our results indicate bortezomib maintenance therapy improves PFS and OS in patients with del(17p). Combined treatment with carfilzomib or elotuzumab and Rd, or pomalidomide with low-dose dexamethasone, can improve the outcomes of RRMM patients with del(17p).

## MATERIALS AND METHODS

### Search strategy

We searched the PubMed, Embase, and Cochrane Library databases, and conference proceedings from the American Society of Hematology, the European Hematology Association, and the American Society of Clinical Oncology. The search terms were as follows: “myeloma” and “del(17p)”. The reference lists from candidate studies and relevant review articles were screened manually. The study was approved by the Institutional Review Board of the General Hospital of Shenyang Military, and was performed in accordance with the Declaration of Helsinki.

### Eligibility criteria

Studies were eligible for inclusion in the meta-analysis if they met all of the following criteria: (1) publication data prior to January 2017; (2) prospective analysis; (3) independent data for del(17p) (not for high-risk cytogenetic abnormalities (CAs): del(17p) and t(4;14)); and (4) provided sufficient information to allow calculation of the overall response rate (ORR), progression-free survival (PFS), and OS.

### Study selection

All abstracts were independently reviewed by two investigators. Full-text screening and independent reviews were performed of eligible studies. Articles meeting the criteria at the full-text review stage were included in the meta-analysis. If multiple publications and/or conference abstracts reported on a single trial population, only the most recent or relevant data were selected for analysis.

### Data collection

The following data were extracted for each study: the first author, publication year, study design, study level, enrollment period, number of patients with and without del(17p), patient characteristics (median age, sex, diagnosis [NDMM or RRMM], median time from diagnosis, median number of prior therapies, and previous regimen of bortezomib, lenalidomide, thalidomide, or auto-HSCT for RRMM patients, treatment details, efficacy (ORR ≥ partial response, PFS, and OS), and length of follow-up.

### Risk of bias in individual studies

The Cochrane Collaborations tool was used to assess the risk of bias in randomized trials [15]. This assessment was conducted by two independent investigators. Differences were resolved through discussion with a third reviewer.

### Data analysis

We calculated the pooled incidence of del(17p) in NDMM and RRMM patients, and the ORR in RRMM patients with and without del(17p) using Review Manager (version 5.3; the Cochrane Collaboration, Oxford, England). A random-effect model was used for all analyses, which incorporated the variability of trial results and provided a more conservative estimate of effect size by producing greater confidence intervals (CIs). Study heterogeneity was assessed using Chi squared tests and the I^2^ statistic. A *P* < 0.10 and an *I*^2^ > 50% was considered significant. Because survival and hazard ratios (HRs) were not calculated at the same time-points in the included studies, we could not calculate a pooled survival estimate. Instead, we generated bar graphs of PFS and OS in NDMM or RRMM patients with or without del(17p) using SPSS version 16.0 (Chicago, Illinois, USA).
